# Supplementary material for: Ground deformation reveals the scale-invariant conduit dynamics driving explosive basaltic eruptions
Source: Nat Commun. 2021 Mar 16;12:1683. doi: 10.1038/s41467-021-21722-2 (PMC7966810; doi:10.1038/s41467-021-21722-2)
Supplement: Supplementary file 1 — Supplementary Information [file 41467_2021_21722_MOESM1_ESM.pdf]

Supplementary Materials for

# Ground deformation reveals the scale-invariant conduit dynamics triggering basaltic explosive eruptions

M. Ripepe*, G. Lacanna, M. Pistolesi, M.C. Silengo, A. Aiuppa, M. Laiolo, F. Massimetti, L. Innocenti, M. Della Schiava, M. Bitetto, F.P. La Monica, T. Nishimura, M. Rosi, D. Mangione, A. Ricciardi, R. Genco, D. Coppola, E. Marchetti, D. Delle Donne.

Correspondence to: maurizio.ripepe@unifi.it

## Supplementary Note 1

### Chronology and fieldwork of the 2019 Stromboli's paroxysms

The Stromboli's mild explosive activity[24,25,52-55] is occasionally interrupted by paroxysms, with only two events recorded in the early 2000s (2003 and 2007). In the summer 2019, Stromboli entered a phase of escalating regular explosive activity that culminated, on 3 July 2019, with a first paroxysmal explosion at 14:45:42 UTC. The explosion lasted several minutes, and led to the formation of a 8 km-high eruptive column. The violent explosion covered the village of Ginostra with ash, lapilli and bombs, and generated a pyroclastic flow along the steep slope of the Sciara del Fuoco (https://www.youtube.com/watch?v=zKZ2WrmqIxc). A large sector of the island burnt almost immediately when the hot pyroclastic fragments reached the vegetation. The partial collapse of the explosive column generated two pyroclastic flows which, impacting the sea surface, generated a tsunami that reached the cost of Italy mainland after only half an hour. Luckily, the tsunami was only 2 meters-high but one people in Ginostra village eventually died trying to escape the rain of hot lapilli and bombs in that sector of the island. Before the 3 July paroxysm, no significant variation in the geophysical and geochemical monitored parameters occurred. Nobody was directly injured, but if the explosion would have occurred only few hours later, hundreds of tourists would have been at the summit areas and hit by the explosion with tragic consequences. After the July 3 paroxysm, the regular Strombolian eruptive activity remained at very high-levels for the following weeks. The paroxysm of July 3 also severely impacted the crater area, which was heavily reshaped by the eruption. During this period oh very-high regular activity, a lava effusion started from the south-western sector of the crater terrace, flowing down the Sciara del Fuoco and reaching the sea.

On 28 August 2019, at 10:17:15 UTC, a second paroxysm occurred from the summit craters, generating a new eruptive plume and covering the summit areas with bombs and lapilli and the village of Stromboli with several centimeter of ash. Also this second paroxysm generated a pyroclastic flow along the Sciara del Fuoco that triggered a tsunami after impacting the sea. The effusive activity ceased completely after a few days, on 31 August 2019, although higher degassing from the crater area persisted for several weeks after the eruption ended.

A few days after the paroxysm of 3 July, scientific personnel involved in this study reached Stromboli to conduct stations maintenance and preliminary fieldwork after the explosion. All the eruptive crisis was followed and covered with shifts in order to have continuous presence of

personnel on the island. During this period, several field investigations were carried out. Among the different activities of sampling and observations around the crater area, a specific work was carried out to have an estimate of the 3 July erupted volume. Measures of loading per unit area of the fallout deposit were obtained along the western coast of Stromboli; total volume was estimated based on single exponential decay law from plots of loading per unit area of fallout deposits versus isomass area according to[56].

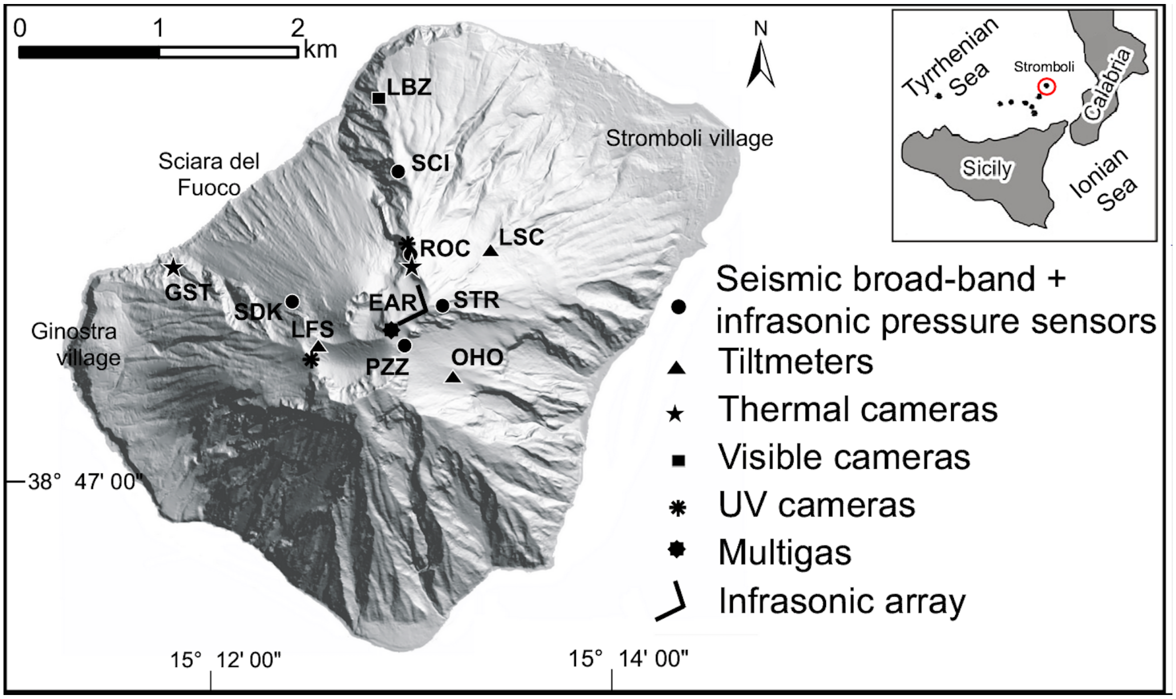


**Supplementary Figure 1.**

Stromboli map and the position of all the geophysical and geochemical stations of the permanent monitoring network operating on 3 July 2019.

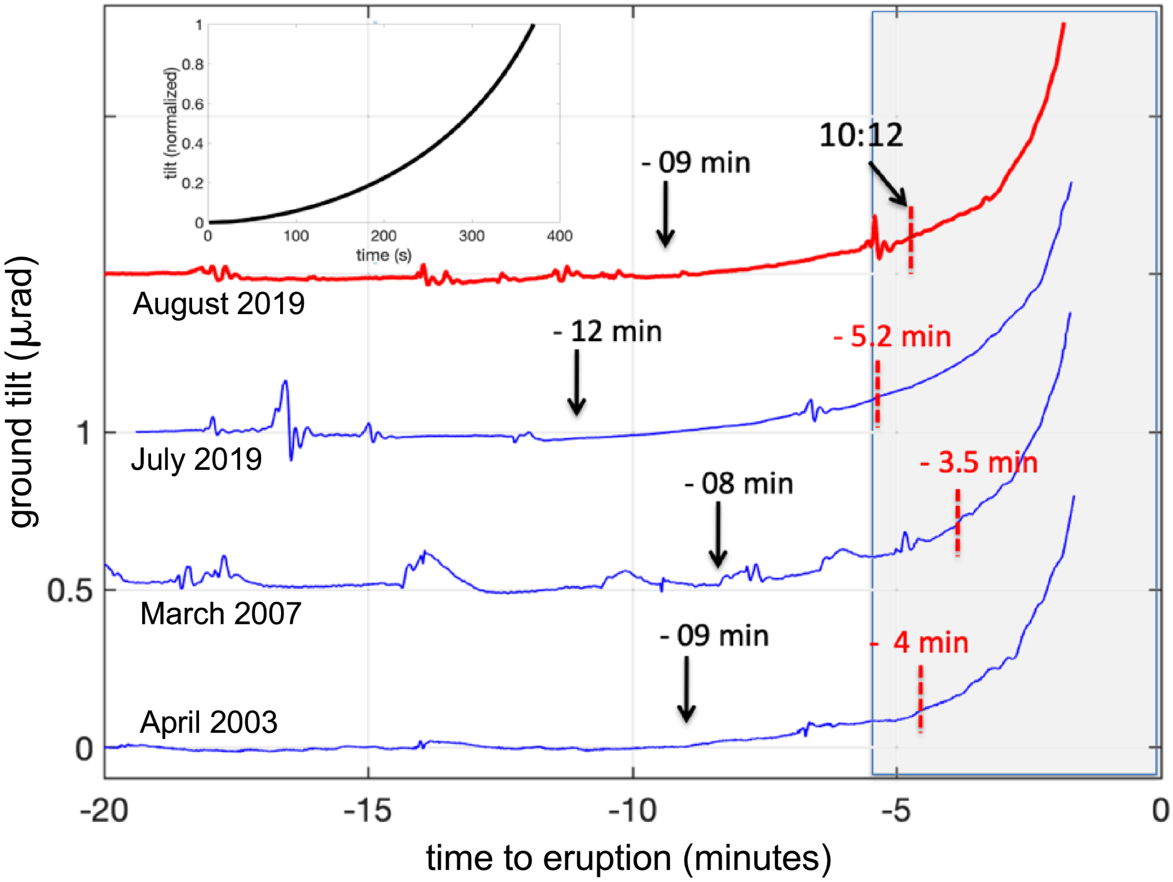


**Supplementary Figure 2.**

Details of the ground deformation preceding the four paroxysms. Inflation becomes sharp and well above the background activity already 8-10 minutes (arrows) before the eruptive onset. Ground tilt has been truncated 2 minutes before the onset and when is only 0.7 μrad large. The gray band indicates the time range within which the Early Warning system can recognize the occurrence of the eruption and, in particular, when (at 10:12) the alert was delivered in case of the 28 August 2019 eruption occurred at 10:17:15. The inset shows the template ground deformation used by the match filter analysis of the Early Warning system to recognize the occurrence of the ground inflation. Black arrows indicate the beginning of the inflation whilst red lines indicate when the Early Warning sends the alerts.

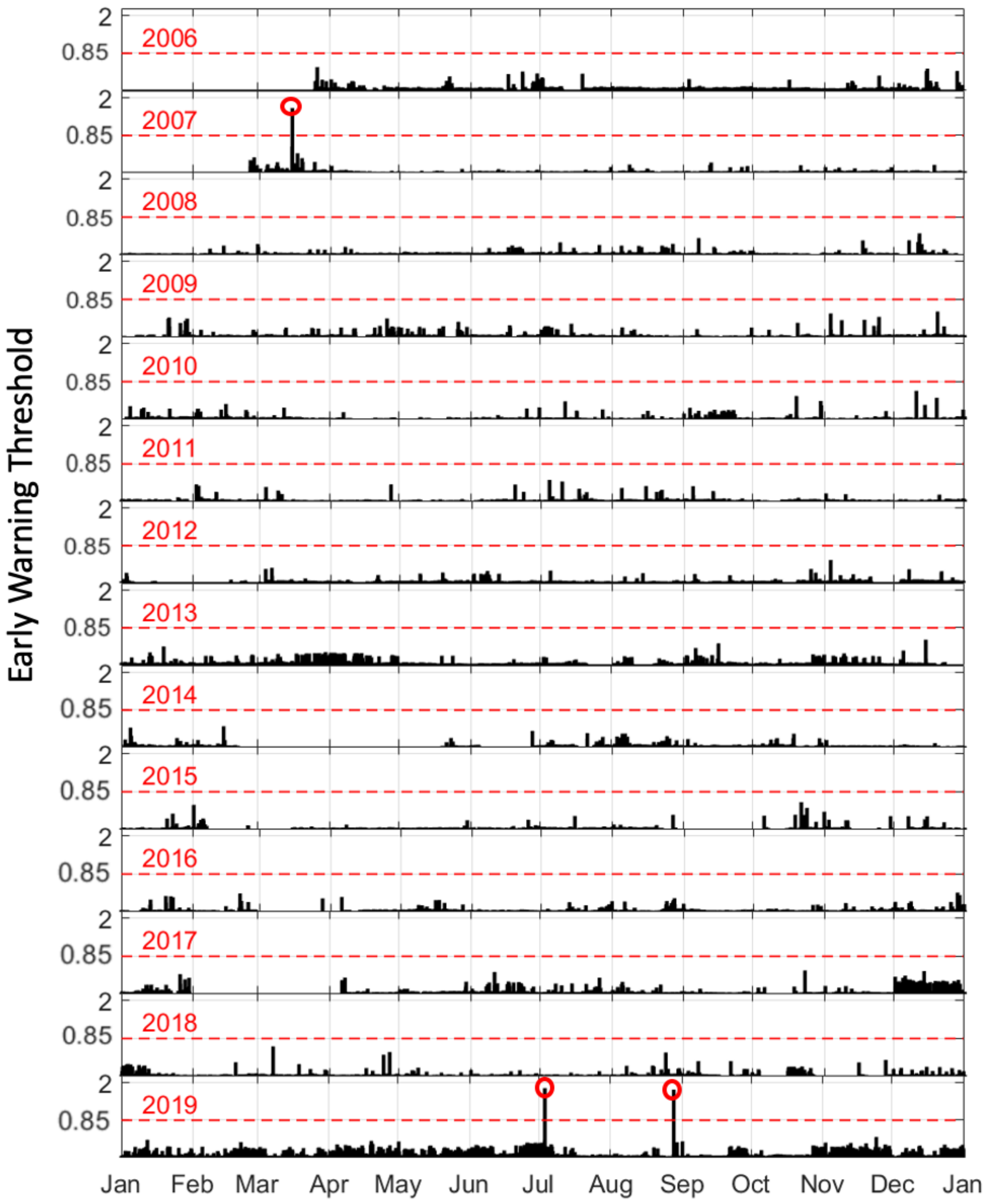


**Supplementary Figure 3.**

Output of the Early Warning algorithm applied to 13 years (2006-2019) of ground tilt measured ad the OHO station (Supplementary Fig. 1). Threshold above 0.85 has been multiplied by 2 for graphical reasons to expand the vertical scale. The threshold of 0.85 is reached only three time, on

15 March 2007, 3 July 2019 and 28 August 2019 and 3 to 4 minutes before the explosion onset (Supplementary Fig. 2). Note that no false alerts are generated by the algorithm in the last 13 years. The system is today fully operative at Stromboli.

**Supplementary References**


52. M. Rosi *et al.*, A case history of paroxysmal explosion at Stromboli: timing and dynamics of the April 5, 2003 event. *Earth Planet. Sci. Lett.* **243,** 594–606 (2006).
53. M. Patrick *et al.*, Strombolian explosive styles and source conditions: insights from thermal (FLIR) video. *Bull. Volcanol.* **69**, 769–784 (2007).
54. A. Bertagnini *et al.*, Volcanology and magma geochemistry of the present-day activity: constraints on the feeding system. In Calvari, S., Inguaggiato, S., Puglisi, G., Ripepe, M., Rosi, M. Eds. Learning from Stromboli. *Am. Geoph., Geoph. Mon.* **182**, 19–38 (2008).
55. L. Francalanci, F. Lucchi, J. Keller, G. De Astis, C. Tranne, Eruptive, volcano-tectonic and magmatic history of the Strom- boli volcano (north-eastern Aeolian archipelago). In: Lucchi, F., Peccerillo, A., Keller, J., Tranne, C.A. Rossi, P. L. (eds) The Aeolian Islands Volcanoes. *Geol. Soc. Lond. Mem.* **37**, 55–81 (2013).
56. D.M. Pyle, The thickness, volume and grain size of tephra fall deposits. *Bull. Volcanol.* **51**, 1–15 (1989).
