# Supplementary material for: Ground deformation reveals the scale-invariant conduit dynamics driving explosive basaltic eruptions
Source: Nat Commun. 2021 Mar 16;12:1683. doi: 10.1038/s41467-021-21722-2 (PMC7966810; doi:10.1038/s41467-021-21722-2)
Supplement: Supplementary file 2 — Description of Additional Supplementary Files [file 41467_2021_21722_MOESM2_ESM.pdf]

Description of Additional Supplementary Files

File Name: Supplementary Movie 1
Description: Synchronized video showing the tiltmeter signal from OHO station of the 3 July 2019 paroxysm, combined with thermal and visible videos from ROC and LBZ stations, respectively. Ground inflation starts at 14:33:48 UTC (blue dashed line) almost 12 minutes before the eruption onset (black line) occurring at 14:45:42 UTC. Red line at 14:40:38 UTC indicates when the alert is given by the Early Warning algorithm. Note the lava overflow occurring at 14:45:02 UTC, 40 seconds before the eruption.
